# Magnetoencephalographic (MEG) brain activity during a mental flexibility task suggests some shared neurobiology in children with neurodevelopmental disorders

**DOI:** 10.1186/s11689-019-9280-2

**Published:** 2019-08-19

**Authors:** Alexandra Mogadam, Anne E. Keller, Paul D. Arnold, Russell Schachar, Jason P. Lerch, Evdokia Anagnostou, Elizabeth W. Pang

**Affiliations:** 1 Institute of Medical Science, Faculty of Medicine, University of Toronto, Toronto, Canada; 2 Neurosciences and Mental Health, SickKids Research Institute, Toronto, Canada; 3 Division of Neurology, Hospital for Sick Children, 555 University Avenue, Toronto, M5G 1X8 Canada; 4 Mathison Centre for Mental Health Research and Education, University of Calgary, Calgary, Canada; 5 Genetics and Genome Biology, SickKids Research Institute, Toronto, Canada; 6 Department of Psychiatry, Faculty of Medicine, University of Toronto, Toronto, Canada; 7 Mouse Imaging Centre, Hospital for Sick Children, Toronto, Canada; 8 Department of Medical Biophysics, Faculty of Medicine, University of Toronto, Toronto, Canada; 9 Holland Bloorview Kids Rehabilitation Hospital, Toronto, Canada

**Keywords:** ASD, ADHD, OCD, MEG, Executive function, Set shifting, TOCS, RBS-R, Corticostriatal projections, Neurodevelopmental disorders

## Abstract

**Background:**

Children with neurodevelopmental disorders (NDDs) exhibit a shared phenotype that involves executive dysfunctions including impairments in mental flexibility (MF). It is of interest to understand if this phenotype stems from some shared neurobiology.

**Methods:**

To investigate this possibility, we used magnetoencephalography (MEG) neuroimaging to compare brain activity in children (*n* = 88; 8–15 years) with autism spectrum disorders (ASD), attention deficit hyperactivity disorder (ADHD) and obsessive-compulsive disorder (OCD), as they completed a set-shifting/mental flexibility task.

**Results:**

Neuroimaging results revealed a similar parietal activation profile across the NDD, groups suggesting a link to their shared phenotype. Differences in frontal activity differentiated the three clinical groups. Brain-behaviour analyses showed a link with repetitive behaviours suggesting shared dysfunction in the associative loop of the corticostriatal system.

**Conclusion:**

Our study supports the notion that NDDs may exist along a complex phenotypic/biological continuum. All NDD groups showed a sustained parietal activity profile suggesting that they share a strong reliance on the posterior parietal cortices to complete the mental flexibility task; future studies could elucidate whether this is due to delayed brain development or compensatory functioning. The differences in frontal activity may play a role in differentiating the NDDs. The OCD group showed sustained prefrontal activity that may be reflective of hyperfrontality. The ASD group showed reduced frontal activation suggestive of frontal dysfunction and the ADHD group showed an extensive hypoactivity that included frontal and parietal regions. Brain-behaviour analyses showed a significant correlation with repetitive behaviours which may reflect dysfunction in the associative loop of the corticostriatal system, linked to inflexible behaviours.

**Electronic supplementary material:**

The online version of this article (10.1186/s11689-019-9280-2) contains supplementary material, which is available to authorized users.

## Introduction

Neurodevelopmental disorders (NDDs) are a heterogeneous group of disorders, characterized by compromised central nervous system development and aberrant brain function [1–3]. The most common NDDs include autism spectrum disorders (ASD), attention deficit hyperactivity disorder (ADHD) and paediatric obsessive-compulsive disorder (OCD). While each of these NDDs has its own distinct clinical phenotype (i.e. social communication impairments and repetitive behaviours in ASD, dysfunctions in attention regulation and hyperactivity in ADHD, and impaired control of obsessive thoughts and behaviours in OCD [1]); they are often co-morbid [4] and share genetic [5–7], neurobiological [8] and cognitive-behavioural characteristics, such as impairments in social perception [9], rigidity and difficulties with attention.

One cognitive characteristic observed in all three groups is that of impaired executive functions, including mental flexibility (MF). Mental flexibility comprises the ability to alter behavioural and thought patterns in response to environmental changes [10, 11] and is essential for adapting to changing surroundings, navigating social interactions and learning in academic and work environments. This crucial cognitive function can be assessed using a set-shifting task, in which participants are asked to match stimuli, with matching criteria shifting every few trials.

Mental flexibility relies on a network of brain regions spanning prefrontal, posterior parietal and insular regions, the basal ganglia and anterior cingulate cortex (ACC; [11–13]), as well as the temporal pole (TP) and pre- and supplementary motor regions in typically developing children (TDC; [14]). Functional neuroimaging studies have investigated the neural correlates of MF in children with ASD [15–17] and adolescents and adults with ASD [18], ADHD [19–21], and OCD [19, 22]. In comparison with TDC, these studies have found differences in brain activity associated with MF within these groups, with one reporting similarities across ADHD and OCD [19], although none have compared all three groups together. Similarly, a diffusion tensor imaging (DTI) study of ASD, ADHD and OCD suggested that there may also be shared structural deficits in all three groups, with ASD and ADHD additionally affected [8]. Together, this growing body of neurobiological and genetic evidence proposes that these NDDs are not separate entities that sometimes co-occur, but in fact, are perhaps part of a spectrum with shared aetiologies and overlapping phenotypes (as discussed by [2]).

Most studies of MF have used fMRI as their method of choice. While this tool is excellent for spatial investigations, it is more limited in its temporal resolution [23]. Magnetoencephalography (MEG) is a neurophysiological modality which tracks neural activity with millisecond accuracy making it an excellent complement to fMRI [24]. Investigations into the fast-paced temporal dimension of brain activity can significantly contribute to our understanding of the dynamics of cognitive processes such as MF, in both typical and non-typical populations. In light of emerging evidence of disrupted connectivity (as measured by high temporal resolution oscillatory synchronization) across multiple areas in the brain during tasks of MF in children with ASD [15, 17, 25], we decided to employ MEG in our investigation of MF in NDDs.

To investigate the neural bases of mental (in) flexibility in children with NDDs, we recruited children with ASD, ADHD and OCD to complete a set-shifting task in the MEG scanner. As there is increasing research suggesting that NDDs may exist along a continuum, we hypothesized that the common behavioural manifestation of cognitive inflexibility across ASD, ADHD and OCD groups may be due to some shared underlying neural substrates, existing along a spectrum. Specifically, based on Ameis et al.’s DTI research, we expected to find greater similarities in activity in ASD and ADHD groups, with both groups more affected than the OCD group.

## Materials and methods

### Participants

We recruited 116 children with an NDD between the ages of 8–15 years. After data cleaning for artefacts, a total of 88 children (38 ASD, 28 ADHD, 22 OCD) were included in our final analyses (see Table 1 for demographics). Participants were recruited through the Province of Ontario Neurodevelopmental Disorders (POND) Network from clinics at the Holland Bloorview Kids Rehabilitation Hospital (ASD) and the Hospital for Sick Children (SickKids; ADHD and OCD) in Toronto. Inclusion criteria were a primary clinical diagnosis of ASD, ADHD or OCD, normal or corrected-to-normal vision, ability to comply with neuroimaging protocols and no contradictions for neuroimaging. Co-morbidities and psychotropic medication use were noted but not excluded.

**Table 1:** Summary of demographic information and neuropsychological assessments

	ASD^1^	ADHD	OCD^2^
Number (*N* = 88)	38	28	22
Age	12.26 ± 2.19 years	12.13 ± 1.89 years	11.58 ± 2.29 years
Male to female	31:7	24:4	14:8
FS-IQ-4/SB-IQ	99 ± 18 (*n* = 36)	98 ± 17 (*n* = 22)	117 ± 18 (*n* = 8)
RBS-R total^3^	30	13	30
SWAN-inattention^4^	5	6	3
SWAN-hyperactivation^4^	4	4	2
TOCS^5^	− 7	− 26	18

^1^28.57% (10/35) of participants with ASD received a secondary diagnosis of ADHD

^2^27.27% (6/22) of participants with OCD received a secondary diagnosis of ADHD and 4.55% (1/22) of participants with OCD received a secondary diagnosis of ASD

^3^Repetitive Behaviour Scale—Revised (total score and number of endorsed items score) [26, 27]

^4^Strengths and Weakness of ADHD Symptoms and Normal Behaviour Rating Scales (inattention and hyperactive sub-measures) [28]

^5^Toronto Obsessive-Compulsive Scale [29]

Upon enrolment, primary clinical diagnoses were confirmed using disorder-specific diagnostic measures: Autism Diagnostic Observation Schedule-2 (ADOS; [30]) and Autism Diagnostic Interview-Revised (ADI-R; [31]) for ASD, Parent Interview for Child Symptoms (PICS; [26]) for ADHD, and the Child Yale-Brown Obsessive Compulsive Scale (CYBOCS; [27]) for OCD.

### Neuropsychological assessments

Full-scale intelligence quotients (FSIQ-2/4: WISC-IV®, WASI-I/-II®; Full IQ: Stanford Binet Intelligence Scales®) and four parent-questionnaires were administered to measure repetitive (Repetitive Behaviour Scale—Revised (RBS-R; [28, 29])), obsessive-compulsive (Toronto Obsessive-Compulsive Scale (TOCS; [32])) and inattentive and hyperactive [Strengths and Weaknesses of ADHD Symptoms and Normal Behaviour Rating Scales (SWAN, inattention and hyperactive sub measures; [33])] behavioural patterns in participants. See Table 1 for group scores.

### Task

To assess MF in our clinical groups, we employed an MEG-compatible Intra-Extra Dimensional Set Shift task (IED-task) previously used in our group to test adults [34] and children [14]. In this task, participants match a target stimulus based on a matching rule that changes every few trials. Participants are required to ‘shift’ to the new rule to have a correct match. There were two types of shifts in our study, ‘extra-dimensional’ and ‘intra-dimensional’, where the former involves a more difficult shift between categories (dimensions), while the latter involves an easier shift, within categories. As the extra-dimensional shift is more difficult, it better taps into the mental processes involved in set-shifting; thus, we present the extra-dimensional results only. From here, this is referred to as the ‘Shift’ condition. See Additional file 1, Section 1-1 for full details.

### Behavioural analyses

Accuracy and reaction time for correct Shift and Non-Shift trials were compared across groups using a linear fixed-effects model in SPSS® (v24), with repeated measures for shift type, age as a covariate and an unstructured repeated covariance type.

### Imaging data acquisition and pre-processing

MEG data were acquired supine in a 151-channel CTF Omega system (MISL, Coquitlam, Canada). Analyses were conducted using SPM12 [35] and FieldTrip [36]. Data were filtered (1–50 Hz) and epoched (− 500–1500 ms). Artefacts were rejected (> 2500 fT) and removed (heart and eye artefacts) using ICA [37, 38]. The data were then averaged, and root mean square (RMS) activity plots, summed over all MEG channels, across time, were generated. See Additional file 1, Sections 1-2 and 1-3 for more imaging and pre-processing details, respectively.

### MEG analyses

Empirical Bayesian beamforming (EBB; [39, 40]) was applied to reconstruct sources (12-mm FWHM Gaussian kernel smoothing) from 50 to 500 ms post-stimulus onset, with sliding overlapping time windows (100 ms wide, 50 ms overlap), resulting in a total of eight windows of interest (i.e. 50–150, 100–200 ms, etc.). Between- and within-group contrasts were conducted using independent samples *t* test [SPM(T)], corrected for multiple comparisons with a modified Bonferroni applied to the *p* value of 0.05 [41]. All results report significant corrected brain activity (*p*_corr_ < 0.05) which was visualized through MRIcron [42].

We first conducted a within-group analysis where, for each group, we used a multifactorial design [43] to contrast the Shift with the Non-Shift condition to identify the brain activity associated with shifting. This generated, for each group, a list of regions with significantly greater activation for the Shift condition within each time window.

To explore differences between groups, a multifactorial design [43] was used to contrast the images based on our hypotheses generated from the literature [8]. We tested the following Shift contrasts: OCD > ASD, OCD > ADHD, ADHD > ASD, and ASD > ADHD.

### Brain-behaviour analyses

To further probe the cross-diagnosis shared neurobiological correlates of mental inflexibility, we investigated brain-behaviour relations collapsed across the group. We used a linear regression model (controlled for age) to test whether the magnitude and/or latency of brain activity predicted behavioural measures (TOCS, RBS-R and SWAN), regardless of clinical group (see Additional file 1, Section 1–4). The TOCS and RBS-R measure obsessive-compulsive and repetitive behaviours, respectively, and the SWAN measures inattentive and hyperactive behaviours. These scales were selected as they quantify the severity of behavioural symptoms that may reflect, and/or contribute to, mental inflexibility.

## Results

### Behavioural results

For accuracy, a significant main effect was observed for ‘Age’ [*F* (1,82) = 4.187, *p* = 0.044] but not for ‘Group’ nor ‘Shift Type’. There were no significant interactions. The average age-adjusted accuracy scores per trial type, for each group, are contained in Table 2 (upper).

**Table 2:** Accuracy and reaction times for the set-shifting task, by clinical group

	Non-shift		Intradimensional (easy) shift		Extradimensional (hard) shift	
	Mean	SE	Mean	SE	Mean	SE
Accuracy						
ASD	93.7%	0.8	93.4%	1.1	91.7%	1.2
ADHD	94.2%	0.9	93.2%	1.3	89.7%	1.4
OCD	93.9%	1.0	92.7%	1.5	90.8%	1.6
Reaction time						
ASD	755 ms	41	834 ms	60	914 ms	43
ADHD	812 ms	48	912 ms	70	948 ms	50
OCD	773 ms	55	865 ms	80	870 ms	58

For reaction time, the fixed effects model revealed a main effect for ‘Shift Type’ [*F* (2,82) = 7.050, *p* = 0.001] only. Post hoc analyses, adjusted for multiple comparisons using the Bonferroni method, revealed that Non-Shift trials were faster than Shift trials [*p* < 0.001]. There were no significant main effects for ‘Group’ and no significant interactions. The mean age-adjusted reaction times by group and trial type can be found in Table 2 (lower).

### MEG results

#### Within-group source level analyses: brain regions involved in set-shifting

**Fig. 1:**
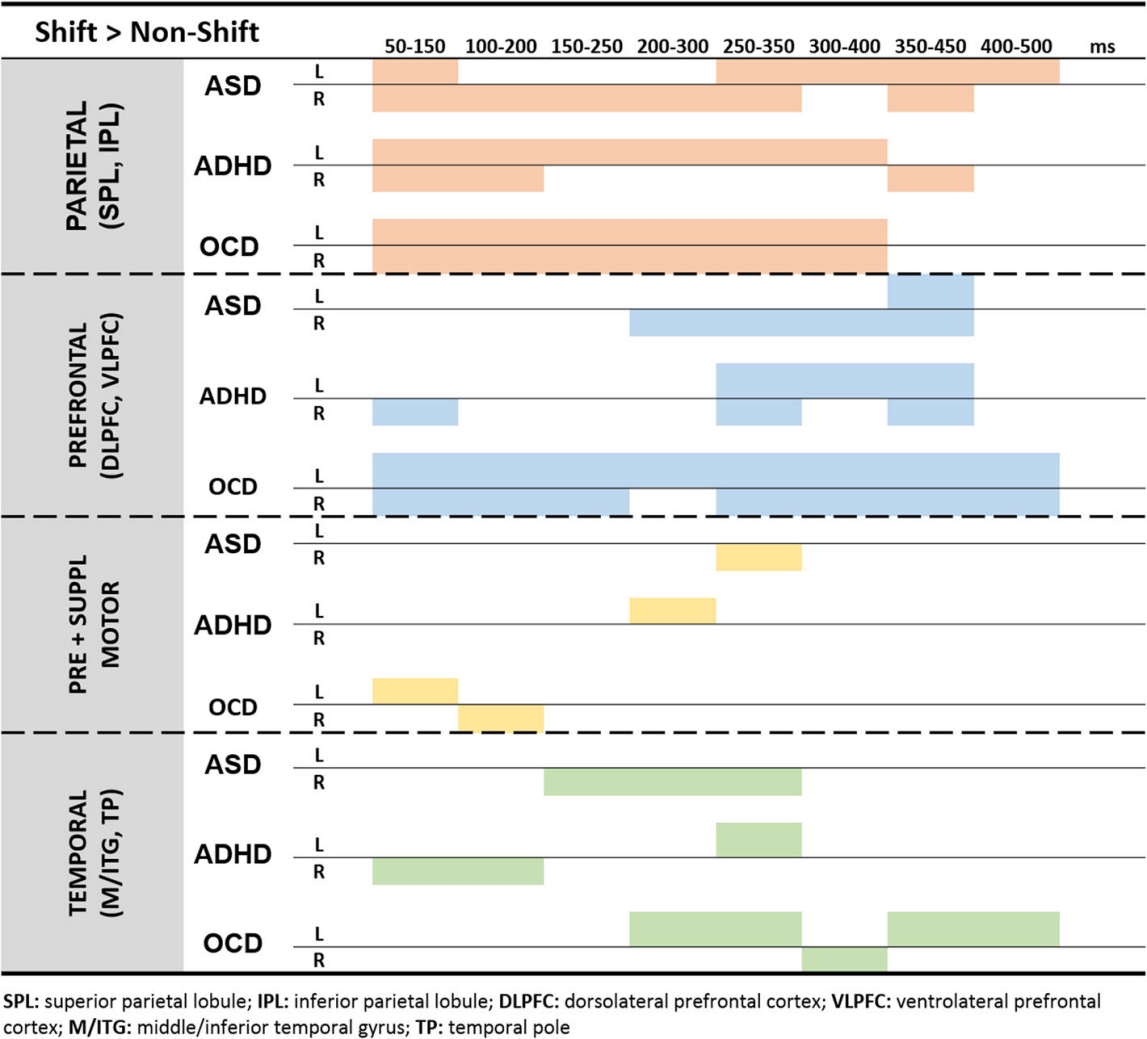
Individual brain activity profiles within each clinical group. For each brain region, the significant activations (*p* < 0.05_corr_) associated with set-shifting are shown, for each clinical group. The activity profiles reveal a shared pattern of sustained parietal activity in all three groups, late and limited prefrontal activity in ASD and ADHD, and sustained frontal activity in OCD. Other regions (pre- and supplemental motor cortices and temporal regions) do not show similarities across groups. SPL superior parietal lobule, IPL inferior parietal lobule, DLPFC, dorsolateral prefrontal cortex, VLPFC ventrolateral prefrontal cortex, M/ITG middle/inferior temporal gyrus, TP temporal pole

Spatiotemporal activity plots identifying brain regions that were significantly more active during set-shifting, for each group, are contained in Fig. 1. For the ASD group, there was sustained activity in parietal lobes involving both inferior and superior lobules (BA 7, 39, 40), with contributions from both hemispheres. Prefrontal activity was dominated by the right inferior frontal gyrus (IFG; BA 44, 45, 47), starting 200 ms post-stimulus onset, until 450 ms. Additional activity was found in the right temporal lobe (BA 37, 38) and the left parahippocampus (BA 36) from 150 to 350 ms, as well as in the right pre- and supplementary motor areas (BA 6) from 250 to 350 ms.

In the ADHD group, similar to the participants with ASD, parietal activity was sustained, although the activity was predominantly in the left hemisphere, in both inferior and superior lobules (BA 7, 39, 40). The right prefrontal regions (BA 10) were briefly active from 50 to 150 ms, and then again later from 250 to 450 ms bilaterally in inferior, middle and superior frontal gyri (BA 8, 10, 46, 47). Similar to ASD, additional activity was found in pre- and supplementary motor areas (BA 6) and the temporal lobe (BA 37), though in the contralateral hemisphere.

Finally, children with OCD also displayed early and sustained bilateral activity in the parietal lobes (BA 7, 39, 40) from 50 to 400 ms post-stimulus onset. Frontal activity was similarly sustained from 50 to 500 ms, also in both hemispheres, across inferior, middle and superior frontal gyri (BA 9, 10, 11, 44, 46, 47). Bilateral pre- and supplementary motor areas (BA 6) were also active early on, with the temporal lobe (BA 37, 38) displaying activity somewhat later, predominantly in the left hemisphere.

#### Between-group contrasts

**Fig. 2:**
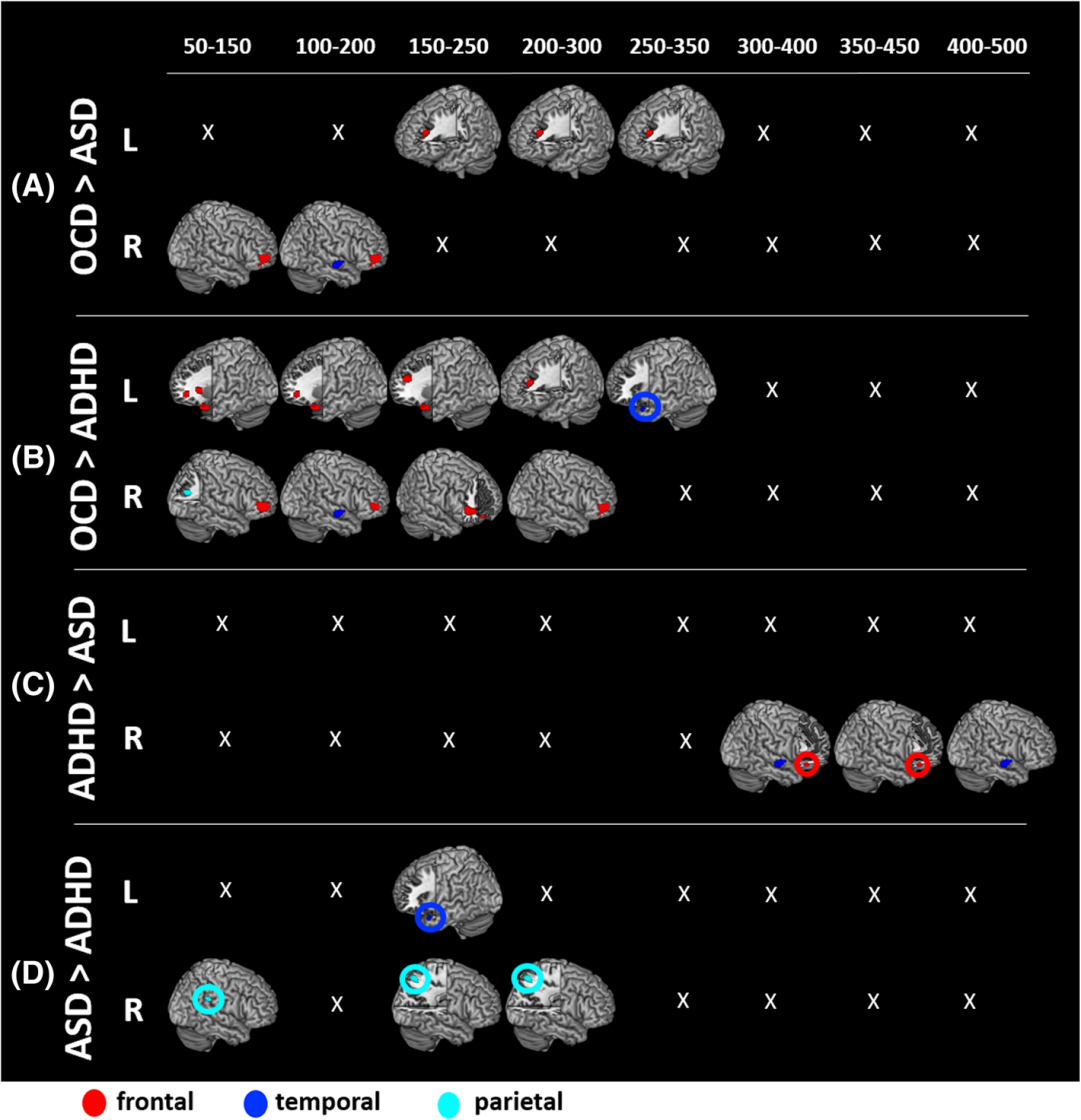
Between-group contrast results, *p* = 0.05_corr_. Between-group contrasts of brain activity associated with mental flexibility (MF). The first two contrasts, **a** OCD > ASD and **b** OCD > ADHD, reveal significantly greater bilateral prefrontal activity in OCD, compared to both ASD and ADHD. The **c** ADHD>ASD and **d** ASD > ADHD contrasts show fewer differences, with ASD showing greater parietal activity and reduced frontal activity compared to ADHD

As per our a priori hypotheses, we conducted four between-group comparisons (contrasting the Shift conditions of two groups at a time; *p*_corr_ < 0.05) where significant differences are displayed in Fig. 2 and delineated in Additional file 1: Table S1. Our a priori hypotheses were that the OCD group would show greater activations compared to both ASD and ADHD groups. The OCD > ASD contrast (Fig. 2a) revealed significantly greater and significantly more sustained (50–350 ms) activity, mainly in frontal regions (middle frontal gyrus, MFG; BA 10) for the OCD group. As well, the OCD group showed a brief (100–200 ms) period of greater activity in the right superior temporal gyrus (STG; BA 22) and the left ACC (BA 25). The OCD > ADHD contrast (Fig. 2b) showed more between-group differences with greater and significantly more sustained (50–300 ms) activity in bilateral prefrontal regions (MFG/ IFG; BA 10, 11, 45, 46, 47) for OCD. Additional differences were found in the parietal (right angular gyrus; BA 39; 50–150 ms) and temporal regions (right STG/BA 22; 100–200 ms and left TP/BA 38; 250–350 ms).

Because we hypothesized that the ASD and ADHD groups would be more similar, we conducted contrasts in both directions. For the ASD > ADHD (Fig. 2c) contrast, a few differences were observed with the ASD group showing greater activity in the right parietal (supramarginal gyrus, SMG/BA 40; 50–150 ms and superior parietal lobule, SPL/BA 7; 150–300 ms) and left temporal (TP/BA 38; 150–250 ms) regions. In the other direction, ADHD > ASD (Fig. 2d), the differences were very sparse. The ADHD group showed greater activations in a late time window in the right IFG/BA 47 (300–450 ms) and right STG/BA 22 (300–500 ms).

### Brain-behaviour analyses

To explore potential relationships between brain regions involved in MF and continuous behavioural measures of clinical symptomology, we regressed peak latency and magnitude during Shift trials with measures of obsession-compulsion (TOCS), repetitive behaviours (RBS-R) and attention/hyperactivity (SWAN), controlling for age. Only the first two measures (TOCS and RBS-R) showed significant relationships (Table 3).

**Table 3:** Significant correlations between brain-behaviour measures

Behavioural measure	Brain region	Brain measure	*β* (beta)	*p*
TOCS^1^	Right SFG	Peak latency	− 0.299	0.008
TOCS	Left IFG	Peak latency	− 0.274	0.015
RBS-R (total)^2^	Left angular gyrus	Amplitude^2^	0.294	0.008

^1^Toronto Obsessive-Compulsive Scale

^2^Repetitive Behaviour Scale—Revised (total score)

For the measure of obsessive-compulsive behaviours captured by the TOCS, we found that the peak latency in two frontal regions, the right superior frontal gyrus (SFG) [*F* (2,79) = 4.084, *p* = 0.021; adjusted *R*^2^ = 0.071; *B* = − 0.102, *p* = 0.008] and left IFG pars triangularis [*F* (2,79) = 3.419, *p* = 0.038; adjusted *R*^2^ = 0.056; *B* = − 0.086, *p* = 0.015], was negatively related with TOCS score. That is, faster peak latencies in these two frontal regions were associated with higher scores, or greater morbidity, regardless of clinical group, on the TOCS scale.

For the measure of repetitive behaviours, we found that a significant linear regression equation predicted RBS-R total scores based on peak power values, extracted during peak two (150–300 ms), [*F* (2,79) = 4.059, *p* = 0.021; adjusted *R*^2^ = 0.070]. Peak power values in the left angular gyrus were significantly positively related to RBS-R total scores, [*B* = 72.025, *p* = 0.008], indicating increased power/activation was associated with greater RBS-R total scores or greater morbidity.

## Discussion

In this study, we used MEG to investigate neural processing involved in a MF task in children with ASD, ADHD and OCD. The ease with which participants completed the task (there were no group differences, with accuracy at approximately 90%) indicated that the children were able to set-shift successfully.

### Similar regions underlie mental flexibility in all groups

In both adults [34] and typically developing control children (TDC; [14]), MF processing has been shown to be subsumed by hubs in bilateral fronto-parietal cortices, the insula and the ACC, with children drawing on additional premotor and temporal lobe regions. Our results concur with this literature as we found that the NDD groups showed recruitment of these brain regions during the task.

### Overlapping activation patterns observed in all groups

The literature in both adults [34] and children [14] suggests that parietal and frontal hubs activate sequentially, without overlap, when completing a set-shifting task. In the current study, our NDD groups showed extensive overlap in the activation pattern of parietal and frontal regions (Fig. 1), with a distinct absence of the sequential progression of activation that is described in the literature. Instead, all three NDD groups showed sustained SPL and inferior parietal lobule (IPL) activation throughout the processing of the task. This sustained posterior parietal activity suggests that all three clinical groups share a strong reliance on the posterior parietal cortices to complete the MF task.

According to the posterior-to-anterior theorem of brain development [44, 45], we know that parietal gyri develop before frontal regions and that often, the posterior parietal cortices play a larger role in mediating executive functions in childhood, until, with increasing age, the frontal areas become more developed and can assume their role in processing executive functions [46–49]. We speculate that our observation of sustained parietal activation that overlaps with the timing of frontal activations suggests that children with NDDs need greater assistance from the parietal regions for their executive functioning. Future studies should investigate whether this observation of high reliance on parietal regions is indicative of delayed brain development or compensation for prefrontal dysfunction.

### Prefrontal activation pattern differentiates OCD

While the NDD groups all showed a similar pattern of sustained parietal activity, the pattern of frontal activations was significantly different between groups, suggesting that this may be a distinguishing feature. Based on the pattern of findings by Ameis et al. in the same NDDs, and in line with our within-group results (Fig. 1), we expected between-group analyses to show the OCD group to less similar to the other two, and we expected ASD and ADHD groups to be more comparable to each other.

Our between-group analyses (OCD > ASD and OCD > ADHD) revealed that children with ASD and ADHD display significantly decreased activity in prefrontal regions compared to children with OCD. The OCD > ADHD contrast revealed the greatest differences, with ADHD showing bilaterally reduced frontal activity across the inferior and middle frontal gyri. These findings are consistent with other functional neuroimaging studies in ADHD which have shown hypofrontality in MF, as well as executive functions more broadly [19–21, 50–54].

The sustained prefrontal activity in OCD may be reflective of hyperfrontality, a functional characteristic that has previously been reported in the OCD literature ([55], for reviews, please refer to [56, 57]), although not necessarily in relation to MF. In adults with OCD, fMRI studies show reduced activation in the classic functional hubs of mental flexibility [58], which related to performance [59]. We were able to hold performance constant between groups; thus, our increased activation may be suggestive of a compensatory mechanism recruited to maintain this high function. Only one study has been conducted in children with OCD [22]. Using fMRI and a set-shifting task, this study reported a decreased hemodynamic response in the left IFG in children with OCD; however, they report greater grey matter density in dorsolateral prefrontal cortex (BA 10), IFG and ACC [22], which may be the neurophysiological mechanism underlying our observation of increased activation in this area. In general, our findings concur with the idea of dysregulation in the prefrontal regions in OCD; however, further studies are needed to understand whether atypical activations are symptomatic of dysfunctional processing or indicative of compensatory function.

### ASD and ADHD differentiated by frontal/parietal abnormalities

We did not have a priori hypotheses as to how the ASD and ADHD groups would compare with each other; thus, we conducted our comparisons in both directions. As we would have predicted from Fig. 2, fewer differences were found in the ASD > ADHD and ADHD>ASD contrasts, compared to the contrasts with the OCD group. However, a definite pattern emerged where we observed significantly reduced right parietal activation in ADHD and reduced right frontal activation in ASD.

In the ADHD group, this reduced parietal activation compared to ASD, and the reduced frontal activation compared to OCD (described above), is consistent with many studies showing that individuals with ADHD have extensive hypoactivity that includes the frontal lobes and the parietal regions, as well as the striatum, insula and ACC [19–21, 51–53].

In ASD, previous studies have shown dysregulations in the connectivity and synchrony of brain regions/networks involved in MF [25], as well as an increased reliance on the parietal lobes in set-shifting compared to TDC [15, 17, 60]. It has been suggested that the brains of individuals with ASD do not develop efficient long-range cortical connections during development, and at the same time, have very well-established short-range connections (U-fibres), resulting in hyper-connectivity of local brain hubs [61–64]. Such a structural organization of the brain would show excessive activation of inter-parietal cortical networks and result in altered function, as in the case of MF. Because of the disruptions in the efficiency of long-range connections, it is possible that the ASD group cannot efficiently recruit their frontal regions, and therefore exercise a greater reliance on their parietal lobes for successful task execution.

### Brain-behaviour analyses

For our first set of MEG analyses (featuring the within- and between-group neuroimaging contrasts), we grouped participants according to their primary diagnosis. While our MEG contrasts did reveal unique MF group-specific brain activity profiles, we also found striking similarities across the NDDs. Increasingly, there is evidence that NDDs are not separate entities that sometimes co-occur, but in fact, may be part of a spectrum with shared aetiologies and overlapping phenotypes [2]. To this end, we conducted brain-behaviour correlations, across all participants, to explore whether measures of neuromagnetic activity in brain regions involved in set-shifting predicted inflexibility-related symptom severity, irrespective of diagnostic group.

We used the RBS-R, TOCS and SWAN as measures of severity of symptomatic behaviours reflective of, and/or contributing to, mental inflexibility. For both the RBS-R and the TOCS, we found that brain activity predicted symptom severity, albeit in opposite directions. Bilaterally in the dorsolateral prefrontal cortices (right SFG and left IFG), we found a negative relation between peak latency and TOCS scores, such that faster peak latencies were significantly related to poorer (increased) TOCS scores. As expected, the OCD group had the greatest morbidity on the TOCS scale. There are a few different ways that we could interpret this finding. It is likely that this brain-behaviour relation is tied to the earlier and generally greater frontal activity observed in the OCD cohort during the MF task. Consistent with the literature, these findings may suggest that the prefrontal regions are hyperactive in children with OCD-like symptoms ([55] for reviews, please refer to [56, 57]). Another possibility is that this brain-behaviour relation indicates that children with NDDs who activate their prefrontal regions earlier exhibit behaviours associated with OCD symptomology, such as impulsive/compulsive behaviours or hyper-performance monitoring. The latter is supported by a study reporting hyperactivity in the MFC during performance monitoring in children with OCD, suggesting greater error-monitoring and a general excessive concern to perform well in this clinical group [55].

In addition to the finding in the prefrontal regions, we also found a significant brain-behaviour relation in the left angular gyrus of the parietal lobe. Increased peak power was positively related with total scores on the RBS-R, meaning that increased peak power was associated with increased repetitive behaviour morbidity. These findings would suggest that increased activity in the left parietal region, a key hub for set-shifting functions, would be associated with increased morbidity of repetitive behaviours. Overall, these brain-behaviour results fit with the between-group comparisons, implying hyperactivity in parietal regions across NDD groups during an MF task. We further speculate that the more a child with an NDD relies on this pronounced involvement/activation of posterior parietal regions during such tasks, the greater their mental inflexibility. Neuropsychological research in adults and children with ASD examining error type on mental flexibility tasks has found a correlation between regressive errors on the task and repetitive behaviour symptomology [65, 66]. While the participants in our study showed extremely high performance, future neuroimaging studies utilizing more challenging tasks could assess atypical brain activity, its interaction with symptomology, error and performance functioning.

In a review paper examining the neurobiology of repetitive behaviours, Langen et al. discuss the different ways in which disruptions to the corticostriatal projections can contribute to inflexible behaviours. Corticostriatal projections are white matter tracts that project from the cortex to the striatum of the basal ganglia, a structure important for motor function and more generally goal-directed behaviour; disruptions to these projections have been associated with repetitive, restrictive and rigid behaviours in various disorders [67, 68]. Repetitive behaviour types can be classified according to their neuroanatomical substrates, and thus, mapped onto distinct corticostriatal loops [69]. Of particular interest is the associative loop, which consists of prefrontal and posterior parietal projections to the striatum, including from the regions in which we found significant brain-behaviour relations [67, 70, 71]. Langen et al. propose that dysfunctions in the associative loop may present behaviourally as impulsivity and/or rigidity. In light of this, it is possible that our brain-behaviour findings are reflective of an underlying corticostriatal dysfunction, impacting the ability of the involved neural regions to operate efficiently, including those that are important in set-shifting, resulting in symptoms of greater inflexibility.

The other corticostriatal loops are thought to be involved with other forms of inflexible behaviour, with the sensorimotor circuit associated with (atypical) stereotypical motor behaviour, and the limbic circuit with motivation-associated facets of behaviour and obsessions and compulsions [69]. Different combinations of variations or pathologies in these loops can create unique and inflexible behavioural patterns across individuals, creating the complexity and overlap in symptoms that are observed in disorders where inflexibility and repetitive behaviours are of issue [72]. Langen et al. propose that these behavioural patterns fall along a continuum, where, for example, a more pronounced abnormality in prefrontal or posterior parietal regions of the corticostriatal system may result in symptomology that would look more like the OCD or ASD cohorts, respectively. In this manner, a child with OCD who displays prefrontal over-engagement during a challenging MF exercise may perform below average, similar to a child with ASD, who displays less and later prefrontal engagement. Even if disruptions occur at different locations, or in different loops, as both children most likely have impairments in their corticostriatal projections, their behavioural output looks similar.

Research into the structural and functional health of frontostriatal regions in NDDs shows ubiquitous atypicalities. Children with ASD and ADHD show decreased white matter integrity along the corticostriatal tracts compared to controls [8]. Other studies have linked atypical frontostriatal (micro) structures to repetitive behaviours in ASD [73–75] and to more errors and more trials to complete a set-shifting task in ADHD [76]. A recent fMRI study in children with ASD and OCD found a relation between increased functional connectivity across frontostriatal regions during resting state and increased morbidity on their measure of repetitive behaviours [77], although this may reverse in adulthood [78].

To further explore the health and function of corticostriatal fibres in children with NDDs, future studies may consider taking a multi-model imaging approach, exploring white matter integrity of the loops and whether any correlations can be found with MF function. As well, it would be of interest to take a blind approach to the analysis and see whether primary diagnoses hold.

### Limitations

There are some important limitations to consider. We are not able to draw direct comparisons between the NDD groups and typically developing children as there was not a control group in this study. Our previous work [14] in typically developing children used the identical task, acquisition parameters and processing pipeline of this study; however, there was an equally balanced sex ratio, whereas in the current study, the cohorts are male-dominated, as would be expected in NDDs [79–81]. Because of the increasing evidence that sex impacts the presentation of the NDDs, we chose not to directly compare the controls to the NDD groups. Future studies should target recruitment of additional males in the control group so as to maintain comparable sex ratios with the NDDs. A second limitation is that while co-morbidities and medications were noted, they were not factored into the analysis. Finally, while we interpret our significant brain-behaviour results, it should be noted that these values account for only a small part of the variance. Future studies, possibly with much larger sample sizes and incorporating an age- and sex-matched control group, should attempt to address these limitations. Despite these limitations, we believe in the value of these findings and hope they may be hypothesis-generating for other groups working in this field.

## Conclusion

In conclusion, while children with ASD, ADHD and OCD behaviourally share the same impairment in MF, using MEG, we found a pattern of similarities and differences in the neurobiological bases supporting this executive function. We observed that the three groups share neurofunctional characteristics in the parietal regions, but differ primarily in the frontal lobes. We observed that the NDD groups showed an absence of sequential brain activations, but instead, they showed sustained parietal activation which overlapped with frontal activation. This finding suggests that the three clinical groups share a delay or irregularity in brain development; a longitudinal study with a control group is recommended to draw firmer conclusions within this domain. ASD and ADHD groups seemed more affected than the OCD with limited and late frontal lobe activations; however, it remains to be seen whether the sustained prefrontal engagement in OCD is typical. Analyses linking brain activity with behavioural symptom measures revealed significant relations between the activity in prefrontal and parietal regions, and morbidity as measured on behavioural scales of repetitive and stereotypical behaviours, as well as obsessive-compulsive behaviours. These, in turn, may be reflective of a dysfunction in the associative loop of the corticostriatal system, which has been associated with inflexible behaviours, and has been found to be affected in children with NDDs. These findings raise the possibility that this neural system may be a target for intervention. Finally, we believe our findings are in line with new thinking that the NDDs exist along a complex continuum, where, despite the differing core phenotypic characteristics of the existing diagnostic groupings, NDDs appear to share some ‘deeper’ facets that become evident when probing genetic or neurobiological underpinnings of the disorders.

## Additional file

Additional file 1: Supplement 1. (PDF 212 kb)

## Data Availability

The dataset supporting the conclusions of this article is available in the Brain-CODE repository (https://www.braincode.ca).
