# Supplementary material for: Magnetoencephalographic (MEG) brain activity during a mental flexibility task suggests some shared neurobiology in children with neurodevelopmental disorders
Source: J Neurodev Disord. 2019 Aug 19;11:19. doi: 10.1186/s11689-019-9280-2 (PMC6701152; doi:10.1186/s11689-019-9280-2)
Supplement: Supplementary file 1 — Supplement 1. (PDF 212 kb) [file 11689_2019_9280_MOESM1_ESM.pdf]

SUPPLEMENT 1.

METHODS

1-1. MEG set-shifting Task

To assess mental flexibility in our clinical groups, we employed an MEG compatible Intra-Extra Dimensional Set Shift task (IED-task) previously used in our group to test adults (1) and children (2). In this task, participants were instructed to match a target stimulus, with one of two other concurrently presented stimuli. There were 36 possible stimuli, comprised of the combinations of 6 shapes and 6 colours. Stimuli were matched according to their shape or colour, but never both. Stimuli were presented in a 'set' where the matching rule was held constant. After 3-8 trials in the set, the rule switched, requiring the participant to also adjust their matching to the 'new' rule. Participants indicated with a button-press whether the target matched the left or right probe stimuli. The task was self-paced (to a maximum of 4 sec) and the inter-stimulus interval varied between 1.0-1.5s. Response times and accuracy were recorded for each matching response. Children with a mean accuracy of <75% were to be excluded from further analyses; no children were excluded.

Switches were either intra-dimensional or extra-dimensional. Intra-dimensional (ID) shifts involved shifts that remained within the dimension of shape or colour (i.e., colour-to-colour or shape-to-shape switch). Extra-dimensional (ED) shifts featured shifts between dimensions (i.e., colour-to-shape or vice versa). ED shifts are considered to be more cognitively demanding than ID shifts.The trial immediately following the 'shift' trial was labelled a 'Non-Shift' trial. In total, the set-shifting task consisted of 50 ID-Shift trials, 50 ED-Shift trials, and 100 Non-Shift trials. Participants practiced the task prior to entering the MEG.

Within the MEG, stimuli were back-projected to a screen located 68cm from participants' eyes, with the stimuli spanning 13° of arc (6.5° each side of the central fixation). For the purposes of this manuscript, only the ED-Shift was discussed, referred to in the body of the text as the 'Shift' condition.

1-2. Imaging Data Acquisition: MEG and MRI Acquisition

MEG data were acquired continuously (600 Hz sampling rate, 0-100 Hz band pass, third-order spatial gradient noise cancellation) in a 151-channel CTF system (CTF Omega, MEG International Services Ltd., Coquitlam, Canada). The children were scanned in a supine position with fiducials placed at the nasion, and left and right pre-auricular points for continuous head-position monitoring. After the MEG recording, fiducials were replaced with radio-opaque markers to allow co-registration with MRI, which served as neuroanatomical underlays for MEG localization analyses. MRI scans were obtained on a 3T Siemens Trio MR scanner (MAGNETOM Tim Trio, Siemens AG, Erlangen, Germany) using a 12-channel head coil (T1-weighted 3D-sagittal MRPRAGE, TR/TE/FA=2300 ms/2.96 ms/90°, GRAPPA=2; FOV=28.8x19.2cm, 240x256 matrix, 192 slices, slice thickness = 1.0 mm isotropic voxels).

1-3. MEG Pre-processing

All MEG analyses were conducted using SPM12 (3) and FieldTrip (4) software packages, in MATLAB R2015a (MathWorks, Sherborn, MA). Initial pre-processing involved filtering (1-50 Hz; Butterworth bandpass filter), epoching (-500ms pre-stimulus onset to 1500ms post-stimulus) and head motion detection. Trials with head motion greater than 5mm within a trial, and 1cm between trials, were removed. Independent component analysis (ICA; 5,6) was applied to individual participants' data in Fieldtrip (4), to remove heartbeat and eye blink artefacts (a maximum of three components were removed per participant). This was followed by artefact

rejection (trials with activity >2500fT were rejected), and data averaging by Shift Type (ID-Shift, ED-Shift, Non-Shift). To have a preliminary look at the data, root-mean-square (RMS) activity plots, summed over all MEG channels, across time, were generated by group and shift type. The RMS plots (see **Supplemental Figure S1A**) show that activity occurred between 50 to 500ms.

1-4. Brain-Behaviour Analyses

To conduct analyses exploring the link between significant brain activations and behavioral measurements, we extracted peak latency and peak power values from core regions known to be involved in mental flexibility from both the adult (1) and child (2) literature. To have a standardized location across all subjects, the coordinates of the regions of interest were obtained from the Automated Anatomical Labeling (AAL) Atlas (7). These regions consisted of the superior frontal gyrus (dorsolateral, medial and orbital), middle frontal gyrus (dorsolateral, medial orbital, and orbital), inferior frontal gyrus (opercular, triangular and orbital), insula, anterior cingulum,-superior parietal lobule, inferior parietal lobule, supramarginal gyrus, angular gyrus and precuneus; all areas were investigated bilaterally. The RMS plots of the Shift condition were used to determine the time windows of extraction. In line with our previous analyses, we only looked at the Shift condition. The time windows were chosen to capture the peaks of activity. More specifically, for frontal regions, we extracted peak latency and power between 50-350ms (see **Supplemental Figure S1B**) and for the parietal regions, we used two windows to encompass the two peaks (50-150ms and 150-300ms; see **Supplemental Figure S1C**).

Of note, we chose not to control for IQ in our correlations as IQ-scores are impacted in neurodevelopmental disorders, thus, statistically they cannot be considered as ‘independent’ covariates, and therefore it is not appropriate to control for them in statistical tests (8).

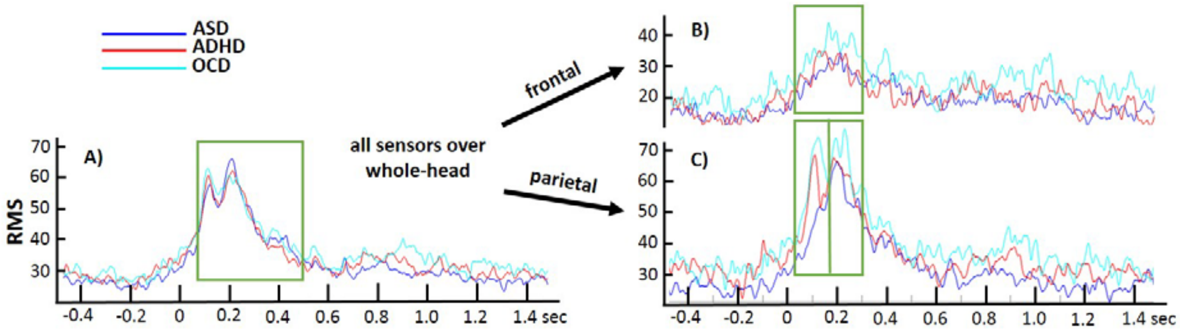

Supplemental Figure S1. Root-mean-square (RMS) activity plots, over time.
A) RMS activity plots over the whole-head, over time for each NDD group. The green box indicates the full time-window where source reconstruction analyses were conducted. B) RMS activity plots over frontal sensors (n=35) for each of the three groups. All three NDD groups display similar peak morphologies with one broad peak. The green box indicates the time window within which peak latency and power were extracted. C) RMS activity plots over parietal sensors (n=35) for each of the three groups. All three NDD groups display similar peak morphologies with two peaks. The green boxes indicate the two time windows within which peak latencies and power were extracted.

Supplemental Table S1. Table of significant differences ($p<0.05_{corr}$) in pair-wise comparisons.

Time (ms)	Laterality	BA	Region	Z	MNI (x,y,z)
			**OCD>ASD**		
50-150	R	10	Inferior/middle frontal gyrus	2.97	42 48 0
100-200	R	10	Inferior/middle frontal gyrus	2.82	40 50 0
	R	22	Superior temporal gyrus	2.79	64 -10 -4
150-250	L	10	Middle/superior frontal gyrus	2.84	-28 44 24
200-300	L	10	Middle frontal gyrus	2.86	-28 40 26
250-350	L	10	Middle frontal gyrus	2.57	-28 40 26
			**OCD>ADHD**		
50-150	R	46	Middle frontal gyrus	3.28	42 44 0
	L	45	Inferior frontal gyrus	3.16	-52 24 10
	L	46	Middle frontal gyrus	2.70	-40 46 0
	L	11	Inferior frontal gyrus	2.90	-20 16 -26
	R	39	Inferior parietal lobule (angular gyrus)	2.60	30 -70 22
100-200	L	11	Inferior frontal gyrus	3.58	-18 16 -26
	R	46	Middle frontal gyrus	3.42	42 40 4
	L	46	Inferior/middle frontal gyrus	2.68	-44 30 16
	R	22	Superior temporal gyrus	2.59	56 2 -6
150-250	L	11	Inferior frontal gyrus, medial face	3.00	-2 38 -18
	R	11	Inferior frontal gyrus, medial face	2.98	6 50 -16
	L	47	Inferior frontal gyrus	2.90	-20 24 -24
	L	10	Middle frontal gyrus	2.93	-28 42 24
	R	46	Inferior/middle frontal gyrus	2.67	44 44 4
200-300	R	10	Inferior/middle frontal gyrus	2.74	8 52 -8
	L	10	Middle/superior frontal gyrus	2.66	-28 40 26
250-350	L	38	Temporal pole	2.59	-44 16 -26
			**ADHD>ASD**		
300-400	R	22	Superior temporal gyrus	3.07	64 -22 -4
	R	47	Inferior frontal gyrus	2.63	48 34 -10
350-450	R	47	Inferior frontal gyrus	2.73	48 30 -10
400-500	R	22	Superior temporal gyrus	2.71	60 -2 -6
			**ASD>ADHD**		
50-150	R	40	Inferior parietal lobule (supramarginal gyrus)	2.57	58 -40 34
150-250	L	38	Temporal pole	2.65	-40 6 -40
	R	7	Superior parietal lobule	2.60	16 -68 56
200-300	R	7	Superior parietal lobule	3.27	14 -70 56

REFERENCES FOR SUPPLEMENT 1

1. Oh A, Vidal J, Taylor MJ, Pang EW. Neuromagnetic correlates of intra- and extra-dimensional set-shifting. Brain Cogn. 2014 Apr;86:90–7.
2. Mogadam A, Keller AE, Taylor MJ, Lerch JP, Anagnostou E, Pang EW. Mental flexibility: An MEG investigation in typically developing children. Brain Cogn [Internet]. 2017 Oct 20 [cited 2017 Oct 25]; Available from: http://www.sciencedirect.com/science/article/pii/S0278262617301197
3. Statistical Parametric Mapping. Wellcome Trust Centre for Neuroimaging London; 2014.
4. Oostenveld R, Fries P, Maris E, Schoffelen J-M. FieldTrip: Open Source Software for Advanced Analysis of MEG, EEG, and Invasive Electrophysiological Data. Comput Intell Neurosci [Internet]. 2010 Dec 23 [cited 2017 Jan 6];2011:e156869. Available from: https://www.hindawi.com/journals/cin/2011/156869/abs/
5. Delorme A, Makeig S. EEGLAB: an open source toolbox for analysis of single-trial EEG dynamics including independent component analysis. J Neurosci Methods [Internet]. 2004 Mar 15 [cited 2016 Dec 10];134(1):9–21. Available from: http://www.sciencedirect.com/science/article/pii/S0165027003003479
6. Kovacevic N, McIntosh AR. Groupwise independent component decomposition of EEG data and partial least square analysis. NeuroImage [Internet]. 2007 Apr 15 [cited 2017 Jan 6];35(3):1103–12. Available from: http://www.sciencedirect.com/science/article/pii/S1053811907000407
7. Tzourio-Mazoyer N, Landeau B, Papathanassiou D, Crivello F, Etard O, Delcroix N, et al. Automated Anatomical Labeling of Activations in SPM Using a Macroscopic Anatomical Parcellation of the MNI MRI Single-Subject Brain. NeuroImage [Internet]. 2002 Jan [cited 2016 Dec 12];15(1):273–89. Available from: http://www.sciencedirect.com/science/article/pii/S1053811901909784
8. Dennis M, Francis DJ, Cirino PT, Schachar R, Barnes MA, Fletcher JM. Why IQ is not a covariate in cognitive studies of neurodevelopmental disorders. J Int Neuropsychol Soc JINS [Internet]. 2009 May [cited 2017 May 13];15(3):331–43. Available from: http://www.ncbi.nlm.nih.gov/pmc/articles/PMC3075072/
